# Transactions of the Chicago Society of Physicians and Surgeons, September 2, 1874

**Published:** 1874-10-01

**Authors:** Ralph E. Starkweather


### TRANSACTIONS OF THE CHICAGO SOCIETY OF PHYSICIANS AND SURGEONS.

### SPECIAL MEETING, SEPTEMBER 2, 1874.

*Reported by Ralph E. Starkweather, M.D.*

THE President, Dr. John Bartlett, in the chair. The meeting had been called by request of several members, in order that the society might hear Dr. Ely M'Clellan, Assistant Surgeon of the U. S. army, speak informally upon the cholera epidemic of 1873. He said that in 1873, Pelikan, and other Russian sanitarians, startled the medical world by declaring that cholera had become endemic in Russia. Recently, Dr. Radcliffe, at a meeting of the British Medical Association, demonstrated the error of these conclusions. He then pointed out upon the map the two lines of travel from India to Europe The north Persian, the route by way of Jelalabad, Herut, Meschid to Asterabad, upon the Caspian sea, across to Baku, thence overland to Tiflis and to Poti, upon the Black sea.

Secondly, the Red sea route, from India, by way of Bombay, down the river Indus to Kurrachee, thence up the Persian gulf via Shiraz and Ispahan to Teheran, the junction of the north and south Persian routes. Kiev, the holy city of Russia, is located on the river Dnieper, and is visited annually by thousands of pilgrims. In 1869 the cholera spread from Kiev, down the river Dnieper to Odessa. It arrived (1870), on the Baltic sea, by the rivers Niemen and Vistula, which are connected with the Dnieper by a series of canals. In 1871 cholera prevailed in Prussia and Austria; in 1872 Austria, Prussia, Italy, France and Holland; in 1873 in Great Britain; in 1873, too, cholera entered New Orleans, from Europe, no less than seventy-three vessels having arrived from infected ports.

The history of an initial case, in May, 1873, was then given, with great clearness and rapidity, of an Ohioan, sick with the cholera on a river steamer, bound from New Orleans to Cincinnati. He died before reaching Memphis, at which place his remains, as well as his garments and soiled clothes, were taken ashore, the body put into a casket and forwarded to his home. The German woman who washed the body died from cholera two days afterward. From these two cases the epidemic originated, and from no others.

Near this city various public works were in progress, canals and railroads. It was in this city (Memphis), that the great outbreak or explosion of cholera took place, (it was not the first point of infection, however,) from which in all directions the disease was carried. The various lines of railroads in the state of Kentucky were pointed out as having numerous towns into which cholera had been brought by commercial travelers, by negroes, or by infected clothing. Then, various counties of Kentucky were mentioned as having peculiarities of the epidemic, due to the remoteness from lines of travel, or to streams of water, or good general hygienic condition. It was in Adair county that the cholera was most malignant, of the counties of that state (Kentucky), especially in the town of Columbia, where, in the rear of a livery stable there was a privy vault, full of the most foul putridity; the ground adjoining it was saturated with the fluid from this pest-hole. The proprietor of the stable rejected all attempts made to have it cleaned. Late in August a negro who had cholera used this privy; from this point of origin, twenty-six persons lost their lives from cholera.

Reference was then made to the Marion county (Ky.) Fair, the Doctor explaining how universal is the custom among the people of attending these gatherings, and said that the Fair held in August of 1873 proved a most disastrous means of general dissemination of cholera through that and all the adjacent counties. He was understood to say that it was in one of the patients who took cholera at this time and place, that there was complete *retention of urine for five and one-half days*, with subsequent recovery.

The depressing influences of a panic were forcibly and vividly illustrated by the cases in Garrard county, Ky. In 1836 there had been a devastating epidemic of cholera in that county, and from that time the people had handed down stories of its ravages and sorrowful scenes. When, therefore, after combating the disease in 1873 as being cholera morbus, it was declared to be Asiatic cholera, the consternation and panic became overwhelming, and the disease very virulent and fatal.

A graceful tribute was paid to the authorities of Lancaster, Garrard county, Ky., for that which had seldom been done elsewhere, namely, paying the physicians who labored to subdue the disease. They also disinfected the districts, and took care of the poor at the public expense. Doctor M'Clellan said that he was anxious to obtain the records of the epidemic in the direct line of the disease; he had no desire to be *ex parte*, or to support or advance any theories, but simply to secure facts. He wished physicians would record their cases, and would be glad to have them give him notes of any, stating the details as minutely as possible, such as the sex, age, color, condition in life, date of attack, date of recovery, date of death, remarks and treatment.

As to the pathology and treatment of cholera, little was said, for lack of time. It was considered to have three stages. The first was the premonitory painless diarrhœa. Cholera is the only malignant disease amenable to treatment in its first stage; this should be promptly treated, else, if it go beyond, the patient is where no human power can help him. In 1873 there was cholera in Mississippi, Alabama, Georgia, Missouri, Kentucky, Arkansas, Ohio, Illinois, Dakota, Minnesota, and, for the first time, it went to the Rocky Mountains. It was most severe in Mississippi, Kentucky, Illinois, and eastern Tennessee.

Dr. Hay said he was glad to have heard these outlines of the progress of cholera; it had modified his opinion; he had been disposed to doubt whether it had truly been the cholera. The information Doctor M'Clellan had given him and allowed him to examine, had removed all his doubts entirely. He could render no more effective service to the profession than the collection of data, such as have been partially given here to-night. In the problem of epidemics there are three essential factors: *First.* The existence of a specific poison, not marsh miasmata, or not it alone. *Second.* Atmospheric and telluric conditions that could be transported or propagated. *Third.* A condition of the individual human system favorable to the reception and development of the poison; if either factor be absent, the problem fails.

We may justly look forward to important results from the work now in progress under the direction of the Doctor.

Dr. Hay moved a vote of thanks of this society be given to Doctor M'Clellan, for the able remarks he has kindly given us this evening. The same was carried with hearty approval.

Dr. I. N. Danforth. Those who have listened attentively must have been convinced of the existence of specific disease germs; they can be traced man by man, from well to well, drain to drain. The Government had certainly assigned the right man to the right place.

Dr. M'Clellan said that he could say one thing, that of all the reports he had yet received, the report of the Board of Health of Chicago was the most comprehensive of any; the facts were clear and to the point, and would give him great assistance; its Microscopical report, by Dr. Danforth, was the only one he had yet found.

Dr. Owens adverted to the fact that this society had appointed a committee last year to study the disease. The Sanitary Superintendent was present and said that the first case of cholera in Chicago, in 1873, occurred on May 24th, at No. 444 Arnold street, in the person of John McFee, a bridge-builder, who had been working near Memphis, and left on account of the cholera. When he arrived in Chicago he had diarrhœa, which remained unchecked, and after a week or ten days developed choleraic symptoms, and proved fatal.

The Society adjourned.

